# Excitability and Threshold Mechanism for Enhanced Neuronal Response Induced by Inhibition Preceding Excitation

**DOI:** 10.1155/2021/6692411

**Published:** 2021-01-18

**Authors:** Hanqing Ma, Bing Jia, Yuye Li, Huaguang Gu

**Affiliations:** ^1^School of Aerospace Engineering and Applied Mechanics, Tongji University, Shanghai 200092, China; ^2^College of Mathematics and Computer Science, Chifeng University, Chifeng 024000, China

## Abstract

Postinhibitory facilitation (PIF) of neural firing presents a paradoxical phenomenon that the inhibitory effect induces enhancement instead of reduction of the firing activity, which plays important roles in sound location of the auditory nervous system, awaited theoretical explanations. In the present paper, excitability and threshold mechanism for the PIF phenomenon is presented in the Morris-Lecar model with type I, II, and III excitabilities. Firstly, compared with the purely excitatory stimulations applied to the steady state, the inhibitory preceding excitatory stimulation to form pairs induces the firing rate increased for type II and III excitabilities instead of type I excitability, when the interval between the inhibitory and excitatory stimulation within each pair is suitable. Secondly, the threshold mechanism for the PIF phenomenon is acquired. For type II and III excitabilities, the inhibitory stimulation induces subthreshold oscillations around the steady state. During the middle and ending phase of the ascending part and the beginning phase of the descending part within a period of the subthreshold oscillations, the threshold to evoke an action potential by an excitatory stimulation becomes weaker, which is the cause for the PIF phenomenon. Last, a theoretical estimation for the range of the interval between the inhibitory and excitatory stimulation for the PIF phenomenon is acquired, which approximates half of the intrinsic period of the subthreshold oscillations for the relatively strong stimulations and becomes narrower for the relatively weak stimulations. The interval for the PIF phenomenon is much shorter for type III excitability, which is closer to the experiment observation, due to the shorter period of the subthreshold oscillations. The results present the excitability and threshold mechanism for the PIF phenomenon, which provide comprehensive and deep explanations to the PIF phenomenon.

## 1. Introduction

A paradoxical phenomenon, the postinhibitory facilitation (PIF) phenomenon [1–3], has been observed in the auditory system, which is related to the inhibition and involved in the sound location [1–5]. The PIF phenomenon states that an additional inhibitory stimulation input with suitable timing can induce the increase of the firing rate [1–3]. For the steady state, the excitatory stimulations with relatively small strength can induce firing with low frequency. After an inhibitory stimulation applied to precede each of the excitatory stimulations, the series of inhibitory–excitatory stimulation pulse pair are formed, which can induce the firing rate increased when the time interval between the inhibitory and excitatory stimulations within each pair is suitable. For a sound in a certain location, the auditory brainstem receives the excitation evoked by the sound from the ipsilateral ear and well-timed inhibition from the contralateral ear [1]. The timing of the excitatory and inhibitory inputs, i.e., the time interval between the inhibitory and excitatory inputs, is a very important factor to induce an action potential or not. Therefore, the PIF phenomenon is very important for the sound location in the auditory system. In addition to the auditory system, for a common neural circuit, a crucial function is the temporal integration of the excitatory and inhibitory inputs. Therefore, the PIF phenomenon is important for the coincidence detection to the inhibitory and excitatory inputs in the common neural circuit. In theory, the PIF phenomenon presents a novel viewpoint different from the traditional viewpoint that the inhibitory stimulations should suppress the firing activity or reduce the firing rate [6–8], which extends the functions of the inhibitory modulations and the contents of the nonlinear dynamics. Based on these studies, the PIF phenomenon and the interval between the inhibitory and excitatory stimulations (interaural time difference induced by a sound) are very important for both neuroscience and nonlinear science. Although the PIF phenomenon is simulated in the theoretical models [2, 3], the theoretical explanations to the PIF phenomenon, especially the excitability and threshold mechanism or the interval between the inhibitory and excitatory stimulations for the PIF phenomenon, remain unclear.

Neuronal excitability is one of the most important characteristics of the nervous system, which mainly describes the ability or activity of the generation of an action potential from the steady state [9–13]. Three types of excitability have been studied in the biological experiments and theoretical models, which are type I, II, and III excitabilities and are defined according to the responses of the resting state to the external stimulations [9, 14, 15]. For type II excitability, firing with nearly fixed period or frequency can be evoked from the resting state by the depolarization stimulation. For type I excitability, with stimulation strength increasing, the firing evoked from the resting state exhibits increasing frequency from nearly zero value. However, the phasic firing with one or several spikes can be evoked from the resting state for type III excitability. In the nonlinear theory, type I and II excitabilities correspond to the saddle-node bifurcation on an invariant cycle (SNIC) and Hopf bifurcation [10, 12, 16, 17], respectively, and no bifurcation appears for the type III excitability [14, 15]. In addition, three different kinds of excitability manifest very different dynamics in multiple aspects, for example, the phase responses to the pulse stimulation or self-feedback [15, 18], the firing patterns to noise or stochastic stimulations [19, 20], the phase resonance curve to the external periodic stimulations [11], and the synchronous behaviors [11, 21, 22], which are involved in different physiological functions. For example, type I excitability and type II excitability have been studied in many kinds of real neurons such as the hippocampal CA1 pyramidal neurons and Dopamine neuron [23–26]. However, type III excitability has been put less attention, for instance, in the subthreshold resonance or coincidence detection of the auditory nervous system [27–32].

However, except for the PIF phenomenon, other multiple paradoxical phenomena, especially induced by the inhibitory or excitatory modulation, have been interpreted with the bifurcations or types of excitability [33–35]. For example, another important example that the inhibitory stimulation can evoke an action potential from the steady state in the nervous system is called postinhibitory rebound (PIR), which is always observed in the nervous system with a hyperpolarization active caution current (Ih) [36, 37]. In theory, the PIR phenomenon has been built a relationship to the Hopf bifurcation or type II excitability [12, 38]. Recently, the PIR phenomenon has been associated with the SNIC bifurcation and type I excitability in a model with Ih current, due to the changes of the threshold surface induced by the Ih current [39]. In addition, an inhibitory self-feedback induces the resting state changed to spiking, which is observed in the dynamic clamp experiment on the interneurons with type II excitability and simulated in the Hodgkin-Huxley (HH) model [40, 41]. In theoretical models, an excitatory self-feedback or memristor can induce the reduction of firing rate of the bursting behavior, which can be interpreted with the bifurcations underling the bursting behavior [42]. Recently, it is discovered that for type II excitability or subcritical Hopf bifurcation in the HH model, the inhibitory or excitatory self-feedback can induce paradoxical phenomena, which is explained with the phase trajectory of the response [41]. For type III excitability, the weak excitatory stimulation with a suitable fast frequency (weak stimulation) can induce the resting state changed to firing behavior, while with a very slow frequency (strong stimulation) cannot induce the firing behavior [15]. The phase trajectory for 3 types of excitability maybe helpful for identification of the threshold mechanism for the PIF phenomenon.

In the present study, we aim to investigate the type of excitability for the PIF phenomenon at first. Unfortunately, we find that the model to simulated PIF phenomenon used in Ref [2] exhibits type III excitability rather than type II and III excitabilities. Therefore, the Morris-Lecar model with 3 types of excitability is used to investigate the PIF phenomenon in the present paper. The excitability and threshold mechanism for the PIF phenomenon and a theoretical estimation to the interval between the inhibitory and excitatory stimulations within a pair for the PIF phenomenon are acquired, which present comprehensive and deep explanations to the PIF phenomenon. Firstly, the PIF phenomenon is simulated for type II and III excitabilities rather than type I excitability. The time interval between the inhibitory and excitatory stimulations for the PIF phenomenon is much shorter for type III excitability, which resembles the experimental observations to a certain extent [1–3]. Secondly, the threshold mechanism for the PIF phenomenon is acquired. For type II and III excitabilities, the inhibitory stimulation induces the subthreshold oscillations around the steady state. During the middle and ending phase of the ascending part and the first phase of the descending part of the oscillations, the threshold to evoke an action potential by an excitatory stimulation becomes weaker, which is the cause for the generation of the PIF phenomenon. For type I excitability, the inhibitory stimulation induces the membrane potential decreased first, and then, the membrane potential recovers to the steady state without oscillations. Therefore, no PIF phenomenon is evoked. Last, a theoretical estimation to the range of the interval between the inhibitory and excitatory stimulations for the PIF phenomenon is presented, which approximates the half period of the subthreshold oscillations. Compared with type II excitability, the period of the subthreshold oscillations becomes much shorter for type III excitability. Therefore, the interval between the inhibitory and excitatory stimulations for the PIF phenomenon becomes shorter for type III excitability.

The rest of the present paper is organized as follows. Materials and Methods, Results and Discussion, and Conclusions are provided in sequence.

## 2. Materials and Methods

### 2.1. Morris-Lecar (ML) Model

The ML model has been widely used to simulated type I, II, and III excitabilities [15, 16, 43–45]. We use the ML model in this study, which reads as

(1) $$\begin{array}{c} C\frac{dV}{dt}=g_{K}w\left(E_{k}-V\right)+g_{\text{Ca}}m_{\infty }\left(E_{\text{Ca}}-V\right)+g_{L}\left(E_{L}-V\right)+I_{\text{app}}+I_{\text{syn}}, \end{array}$$

(2) $$\begin{array}{c} \frac{dw}{dt}=\frac{\phi \left(w_{\infty }-w\right)}{\tau_{w}}, \end{array}$$

where the variable *V* is the membrane potential and the variable *w* represents the recovery variable of the K^+^ channel. The parameter *C* is the membrane capacitance; *g*_*K*_*w*(*E*_*k*_ − *V*), *g*_Ca_*m*_∞_(*E*_Ca_ − *V*), and *g*_*L*_(*E*_*L*_ − *V*) are the K^+^, Ca^2+^, and leakage currents, respectively; *I*_app_ is the external applied current; and *I*_syn_ is the synaptic current to stimulate the external stimulation to the neuron. The parameters *g*_*K*_, *g*_Ca_, and *g*_*L*_ are the K^+^, Ca^2+^, and leakage conductances, respectively; *E*_*k*_, *E*_Ca_, and *E*_*L*_ are the corresponding equilibrium potentials. The parameter *ϕ* is the reference frequency. The functions *m*_∞_, *w*_∞_, and *τ*_*w*_ are, respectively, provided as follows: *m*_∞_ = (1 + tanh((*V* − *V*_1_)/*V*_2_))/2, *w*_∞_ = (1 + tanh((*V* − *V*_3_)/*V*_4_))/2, and *τ*_*w*_ = 1/(cosh((*V* − *V*_3_)/2*V*_4_)), where *V*_1_, *V*_2_, *V*_3_, and *V*_4_ are the tuning parameters.

### 2.2. Parameter Values

The parameter values for the three types of excitability in the ML model are listed in Table 1.

### 2.3. Synaptic Current Model

Similar to Ref [2], the synaptic current *I*_syn_ is used to simulate the inhibitory and excitatory stimulations, which is described as follows:

(3) $$\begin{array}{c} I_{\text{syn}}=g_{\text{ex}}\left(V-E_{\text{ex}}\right)+g_{\text{inh}}\left(V-E_{\text{inh}}\right),\\ \begin{array}{c} g_{\text{ex}}=G_{\text{ex}}\left(\frac{t-t_{0\text{ex}}}{\tau_{\text{ex}}}\right)\exp\left(1-\frac{t-t_{0\text{ex}}}{\tau_{\text{ex}}}\right)H\left(t-t_{0\text{ex}}\right), \end{array}\\ \begin{array}{c} g_{\text{inh}}=G_{\text{inh}}\left(\frac{t-t_{0\text{inh}}}{\tau_{\text{inh}}}\right)\exp\left(1-\frac{t-t_{0\text{inh}}}{\tau_{\text{inh}}}\right)H\left(t-t_{0\text{inh}}\right), \end{array}\\ \begin{array}{c} H\left(t-t_{0}\right)=\left\{\begin{array}{c} 0,t\leq t_{0}\\ 1,t>t_{0} \end{array}\right., \end{array} \end{array}$$

where *t*_0ex_ and *t*_0inh_ represent the application time of the excitatory stimulation and inhibitory stimulation, respectively. *G*_ex_ and *G*_inh_ are the intensity of the excitatory and inhibitory stimulations, respectively, and the unit is mS/cm^2^. The parameters *τ*_ex_ and *τ*_inh_ are the time constant of the excitatory and inhibitory synapses, respectively, and the unit is ms. *E*_ex_ and *E*_inh_ are the reversal potential for the excitatory and inhibitory synapses, respectively, and the unit is mV.

The synaptic parameter values are as follows: *E*_ex_ = −10 mV, *E*_inh_ = −66.5 mV, *τ*_ex_ = 3 ms, and *τ*_inh_ = 10 ms for type I excitability; *E*_ex_ = −10 mV, *E*_inh_ = −66.5 mV, *τ*_ex_ = 3 ms, and *τ*_inh_ = 1 ms for type II excitability; and *E*_ex_ = −10 mV, *E*_inh_ = −96.5 mV, *τ*_ex_ = 0.25 ms, and *τ*_inh_ = 1 ms for type III excitability. The values of the synaptic parameters are chosen according to Ref [2] and different dynamics of 3 types of excitability, which is explained in the last paragraph of the present paper.

### 2.4. Calculation Methods

The ML model is integrated using the fourth-order Runge–Kutta method with a fixed time step of 0.01 ms. The bifurcation diagrams and dynamics in the phase plane are calculated with the software package XPPAUT (http://www.math.pitt.edu/bard/xpp/xpp.html) [46].

## 3. Results and Discussion

### 3.1. The PIF Phenomenon for Type II and III Excitabilities Instead of Type I Excitability

#### 3.1.1. One Stimulation Pair

**Figure 1:**
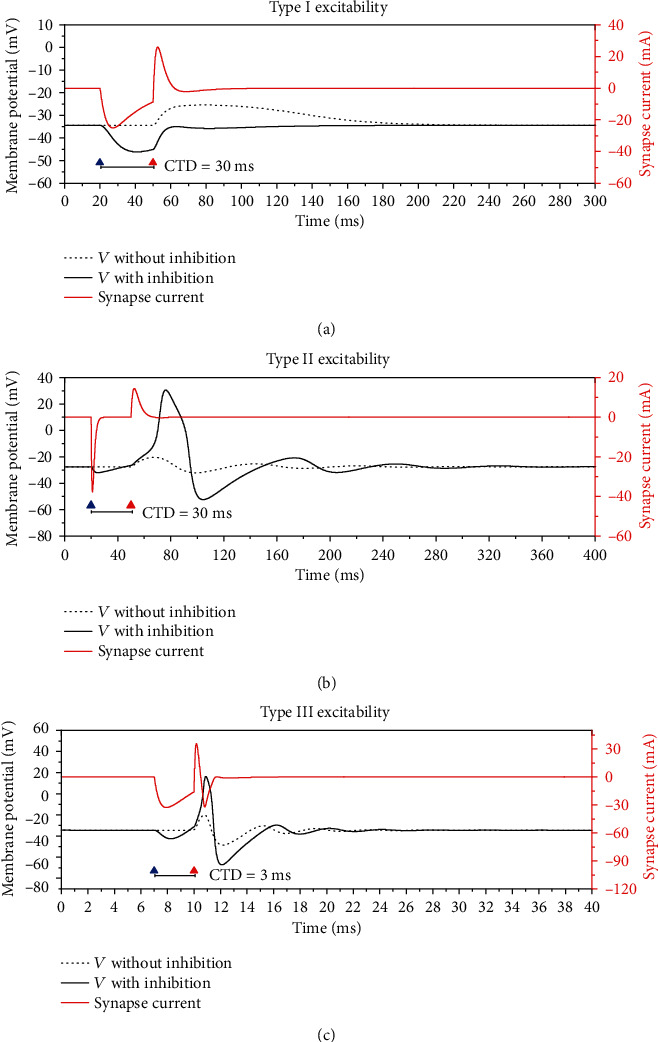
The responses of the membrane potential. Black dashed curve represents the response to an excitatory stimulation (red triangle) and black solid curve to a pair of inhibitory stimulation (blue triangle) and excitatory stimulation. The red curve represents the inhibitory and excitatory stimulations. (a) The PIF phenomenon does not appear for type I excitability. Inhibition stimulation (*G*_inh_ = 1 mS/cm^2^) at *t* = 20 ms and excitation stimulation (*G*_ex_ = 1.1 mS/cm^2^) at *t* = 50 ms. (b) The PIF phenomenon for type II excitability. Inhibition stimulation (*G*_inh_ = 1 mS/cm^2^) at *t* = 20 ms and excitation stimulation (*G*_ex_ = 1 mS/cm^2^) at *t* = 50 ms. (c) The PIF phenomenon for type III excitability. Inhibition stimulation (*G*_inh_ = 0.6 mS/cm^2^) at *t* = 7 ms and excitation stimulation (*G*_ex_ = 2.5 mS/cm^2^) at *t* = 10 ms.

For type I excitability, an excitatory synaptic stimulation (*G*_ex_ = 1.1 mS/cm^2^) applied at the timing corresponding to the red triangle can induce the increase first and then the decrease of the membrane potential and the recovery to the steady state at last, as depicted by the black dashed curve in Figure 1(a). No action potential is evoked due to the relatively weaker excitatory stimulation. If both the excitatory stimulation (*G*_ex_ = 1.1 mS/cm^2^, at red triangle) and an inhibitory stimulation (*G*_inh_ = 1 mS/cm^2^, at blue triangle) preceding the excitatory stimulation with time interval 30 ms are applied, the membrane potential becomes lower than the dashed curve, as shown by the solid black curve, which is consistent with the traditional viewpoint that the electronic activity is suppressed by the inhibitory stimulation. The red curve represents the stimulation of the inhibitory and excitatory synapse. To be consistent with the experiment [1], the time interval between the excitation and inhibition stimulation in a pair is called the composite timing delay (CTD), as shown in Figure 1. A positive CTD means that the inhibitory stimulus precedes the excitatory stimulus.

For the steady state of type II excitability, an excitatory stimulation (*G*_ex_ = 1 mS/cm^2^) (red triangle) cannot induce an action potential, as depicted by the black dashed curve in Figure 1(b). Different from type I excitability, the membrane potential exhibits subthreshold oscillations with an intrinsic period. A pair of the inhibitory (*G*_inh_ = 1 mS/cm^2^, blue triangle) and excitatory stimulation (*G*_ex_ = 1 mS/cm^2^, red triangle) with CTD 30 ms can evoke an action potential, as shown by the solid black curve, which shows that the PIF phenomenon is evoked. The inhibitory and excitatory stimulation is depicted by the red curve. The membrane potential decreases during the inhibitory stimulation and then increases after the inhibitory stimulation and before the excitatory stimulation. During the excitatory stimulation, the membrane potential further increases to a large extent to form an action potential. After the action potential, the membrane potential recovers to the steady state via the subthreshold oscillations.

The steady state for type III excitability exhibits characteristic similar to that of the type II excitability to a larger extent, as shown in Figure 1(c). Different from type II excitability, the period of the subthreshold oscillations for type III excitatory is much shorter, resulting in the much shorter CTD for the PIF phenomenon correspondingly. The CTD for type III excitability shown in Figure 1(c) is 3 ms. The results for 3 types of excitability imply that the subthreshold oscillations and the intrinsic period of the subthreshold oscillation are related to the PIF phenomenon.

#### 3.1.2. Multiple Pairs of Stimulations: Time Interval between Two Successive Pairs Is Fixed

**Figure 2:**
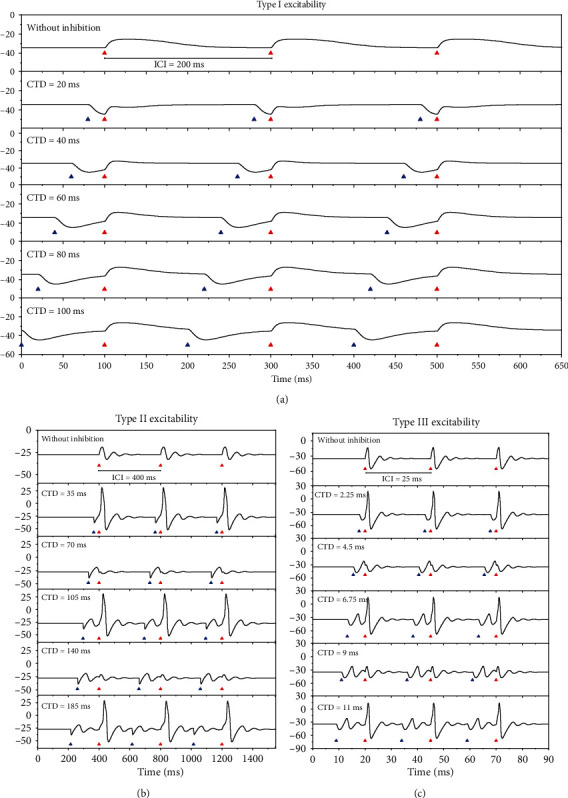
The responses of the membrane potentials to the stimulation pairs of the inhibition (blue triangles) and excitation (red triangles) for the three types of excitability. (a) Type I excitability. The ICI is fixed at 200 ms and *G*_ex_ = 1.1 mS/cm^2^. *G*_inh_ = 0 mS/cm^2^ for the first panel, and the CTDs are 20, 40, 60, 80, and 100 ms from the 2nd to the last rows, respectively, and *G*_inh_ = 0.8 mS/cm^2^. (b) Type II excitability. The ICI is fixed 400 ms and *G*_ex_ = 1.1 mS/cm^2^. *G*_inh_ = 0 mS/cm^2^ for the first panel, and the CTDs are 35, 70, 105, 140, and 185 ms from the 2nd to the last rows, respectively, and *G*_inh_ = 3.1 mS/cm^2^. The PIF phenomenon appears in the 2nd, 4th, and last panels. (c) Type III excitability. The ICI is 25 ms and *G*_ex_ = 3 mS/cm^2^. *G*_inh_ = 0 mS/cm^2^ for the first panel, and CTDs are 2.25, 4.5, 6.75, 9, and 11 ms from the 2nd to the last rows, respectively, and *G*_inh_ = 1.1 mS/cm^2^. The PIF phenomenon appears in the 2nd, 4th, and last panels. The figures show the first three pairs of the stimulations.

To simulate the experiment in Ref [1], the stimulations containing multiple pairs of the inhibitory (blue triangles) preceding the excitatory (red triangles) stimulation pulses are used, as shown in Figure 2. The time interval between the two successive excitatory stimulation pulses, i.e., the interval between two successive pairs, is called inter-click-interval (ICI) in the experiment [1], as shown in Figure 2. In a trial of the stimulation series, both the ICI and the CTD are fixed. The responses of the membrane potentials to the stimulations for the three types of excitability are shown in Figure 2.

The results for type I excitability are shown in Figure 2(a). For the subthreshold excitatory stimulations (red triangles) with ICI 200 ms and *G*_ex_ = 1.1 mS/cm^2^, the subthreshold membrane potentials are evoked, as depicted in the top panel of Figure 2(a). After introducing the inhibitory stimulations (blue triangles) preceding the excitatory stimulations to form pairs with CTD 20 ms, 40 ms, 60 ms, 80 ms, and 100 ms, no action potentials are evoked, as illustrated in the 2nd, 3rd, 4th, 5th, and last panels of Figure 2(a). The result implies that it is difficult to evoke the PIF phenomenon for type I excitability.

In Figure 2(b), type II excitability exhibits very different results. For the subthreshold excitatory stimulations (red triangle) with ICI 400 ms and *G*_inh_ = 3.1 mS/cm^2^, the subthreshold oscillations of the membrane potentials are evoked, as illustrated in the top panel of Figure 2(b). After the inhibitions (blue triangles) are applied to precede the excitatory stimulations (red triangles) with CTD 35 ms, 105 ms, and 185 ms, action potentials are evoked, and each pair of inhibition-excitation stimulation evokes an action potential, as shown in the 2nd, 4th, and last panels of Figure 2(b). However, no action potentials are evoked for CTD 70 or 140 ms, as depicted in the 3rd and 5th panels of Figure 2(b). The result shows that the PIF phenomenon is evoked at the proper CTD values. Compare the 1st and 2nd panels of Figure 2(b), CTD 35 ms is shorter than one period of the subthreshold oscillations. Similarly, 105 ms is between 1 and 2 periods and 185 ms between 2 and 3 periods of the subthreshold oscillations.

For type III excitability, the PIF phenomenon also appears for the proper CTD values, as depicted in Figure 2(c) (*G*_ex_ = 3 mS/cm^2^). For example, when ICI is 25 ms, the PIF phenomenon is evoked for CTD 2.25 ms (2nd panel), 6.75 ms (4th panel), and 11 ms (the last panel), which corresponds to 0-1, 1-2, and 2-3 periods of the subthreshold oscillations, respectively, while is not evoked for CTD 4.5 ms (3rd panel) and 9 ms (5th panel).

**Figure 3:**
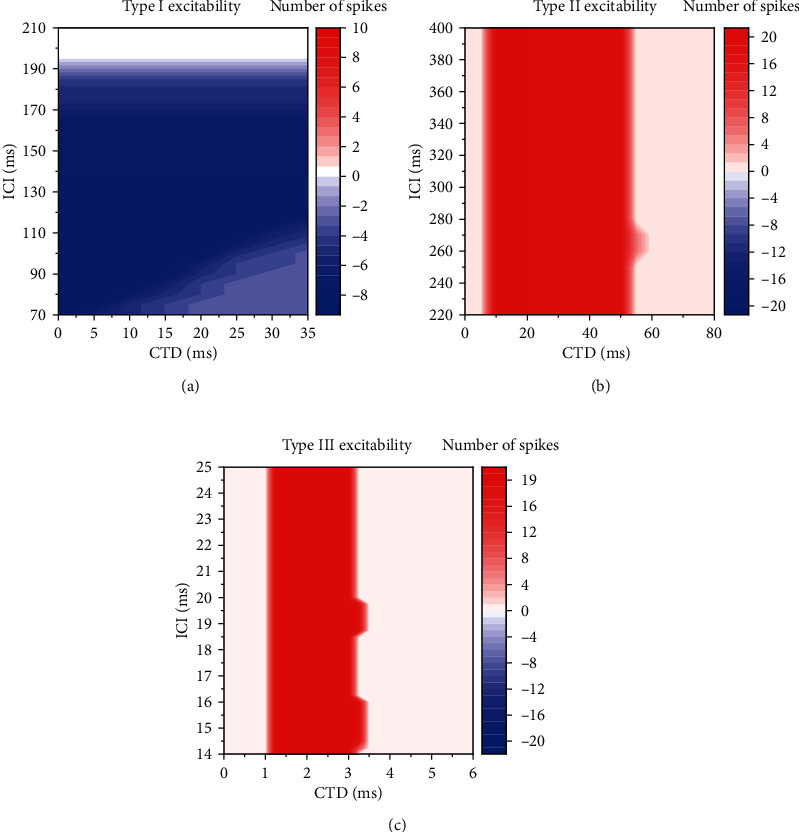
The changes of the firing rate after introducing the inhibitory stimulations in the (CTD, ICI) plane for three types of excitability. (a) Type I excitability with *G*_ex_ = 1.1 mS/cm^2^ and *G*_inh_ = 0.8 mS/cm^2^; (b) type II excitability with *G*_ex_ = 1.1 mS/cm^2^ and *G*_inh_ = 3.1 mS/cm^2^; (c) type III excitability with *G*_ex_ = 3 mS/cm^2^ and *G*_inh_ = 1.1 mS/cm^2^.

The firing rate in the (CTD, ICI) plane for a fixed *G*_inh_ subtracting the firing rate for *G*_inh_ = 0 mS/cm^2^ is acquired, as depicted in Figure 3. The results for type I excitability with *G*_inh_ = 0.8 mS/cm^2^, for type II excitability with *G*_inh_ = 3.1 mS/cm^2^, and for type III excitability with *G*_inh_ = 1.1 mS/cm^2^ are depicted in Figures 3(a)–3(c), respectively.

For type I excitability, the firing rate in the (CTD, ICI) plane decreases as the inhibitory stimulation is introduced (*G*_inh_ = 0 increases to *G*_inh_ = 0.8 mS/cm^2^ with *G*_ex_ = 1.1 mS/cm^2^), as depicted in Figure 3(a), which shows that the firing rate decreases (blue and white) after introducing the inhibitory stimulation.

For type II excitability (*G*_ex_ = 1.1 mS/cm^2^), after introducing the inhibitory stimulation (*G*_inh_ = 0 mS/cm^2^ is changed to *G*_inh_ = 3.1 mS/cm^2^), the firing rate increases, and the PIF phenomenon appears in the red regions, i.e., CTD is within (8.5 ms, 51.5 ms), as shown in Figure 3(b). Such a window of CTD for the PIF phenomenon is called PIF window in Refs [1–3], i.e., the PIF window is CTD within (8.5 ms, 51.5 ms). In the red region shown in Figure 3(b), the PIF phenomenon is independent of the ICI values.

The results for type III excitability resemble those of type II excitability to a large extent, as depicted in Figure 3(c). The PIF phenomenon appears in the red region, and the PIF window is CTD within (1.1 ms, 3.1 ms). To be consistent with experiments [1, 2], the shortest CTD window is studied in the present paper. Other CTD windows with longer values implied in Figure 2 are not studied in the present paper.

**Figure 4:**
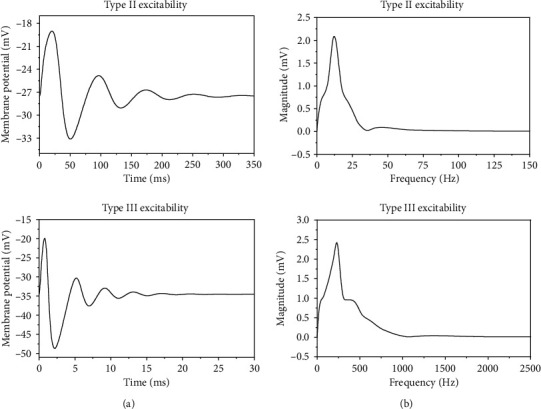
The membrane potential (a) and the corresponding spectrum (b). (a1, a2) Type II excitability. The subthreshold oscillations corresponding to the dashed curve in Figure 1(b). (b1, b2) Type III excitability. The subthreshold oscillations corresponding to the dashed curve in Figure 1(c).

The damping oscillations of the subthreshold membrane potentials evoked from the stable focus for type II excitability and type III excitability is shown in Figure 4(a1 and b1). With the Fast Fourier Transform (FFT), the spectrum of the subthreshold oscillations is acquired, as depicted in Figure 4(a2 and b2), respectively. The intrinsic frequency for type II excitability is about 12.0 Hz, correspondingly; the intrinsic period is around 83.3 ms (*T*_1_), as shown in Figure 4(a2). In Figure 3(b), the CTD for the PIF phenomenon is within 8.5 ms and 51.5 ms, which contains half of the intrinsic period (*T*_1_/2 ≈ 41.7 ms). The width of the PIF window is about 51.5 ms − 8.5 ms = 43 ms, which approximates *T*_1_/2. The intrinsic frequency for type III excitability is about 226.9 Hz, as depicted in Figure 4(b2), and the intrinsic period of type III excitability is around 4.4 ms (*T*_2_). As shown in Figure 3(c), the PIF phenomenon for type III excitability appears for CTD between 1.1 and 3.1 ms, which contains half of the intrinsic period (*T*_2_/2 ≈ 2.2 ms). The PIF window is about 3.1 ms − 1.1 ms = 2.0 ms, which nearly equals to *T*_2_/2. Therefore, for both type II and III excitabilities, the PIF phenomenon is closely related to the period of subthreshold oscillations of the membrane potentials, and the width of the PIF window approximates half period of the subthreshold oscillations.

#### 3.1.3. Random Values for ICIs

**Figure 5:**
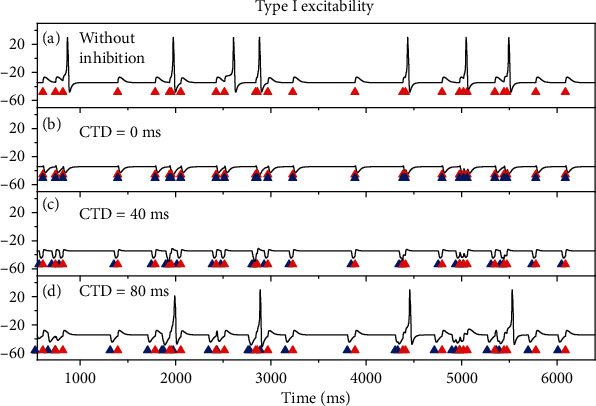
The membrane potentials during the random stimulations for type I excitability (*G*_ex_ = 1  mS/cm^2^). (a) Some random excitatory stimulations (red triangles) can evoke action potentials; *G*_inh_ = 0.0 mS/cm^2^. Inhibitory stimulations (blue triangles) with *G*_inh_ = 0.8 mS/cm^2^ suppress the action potentials in (a): (b) CTD 0 ms; (c) CTD 40 ms; (d) CTD 80 ms.

**Figure 6:**
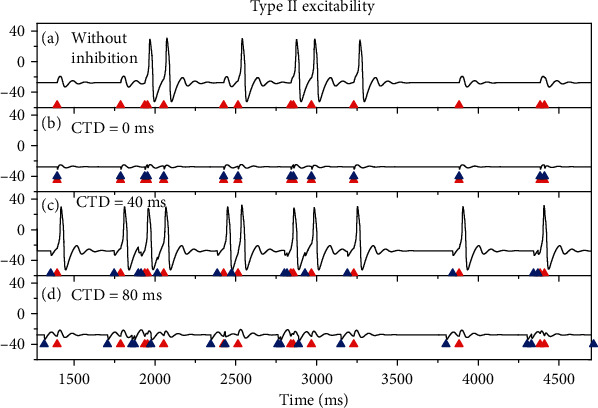
Reponses of the membrane potentials induced by the random stimulations for type II excitability (*G*_ex_ = 1.1 mS/cm^2^). (a) Action potentials induced by some random excitatory stimulations (red triangles) when *G*_inh_ = 0  mS/cm^2^. Inhibitory stimulations (blue triangles) with *G*_inh_ = 1.4 mS/cm^2^ are applied: (b) CTD 0 ms. No PIF; (c) CTD 40 ms. PIF; (d) CTD 80 ms. No PIF.

**Figure 7:**
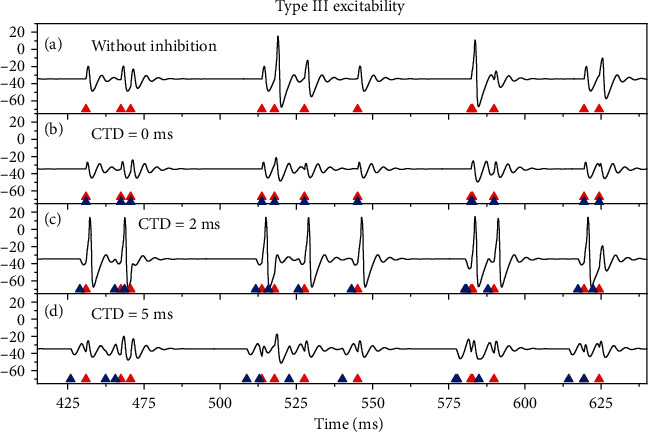
The membrane potentials evoked by the random stimulation for type III excitability (*G*_ex_ = 2.5 mS/cm^2^). (a) Some random excitatory stimulations (red triangles) with *G*_inh_ = 0.5 mS/cm^2^ can evoke action potentials when *G*_inh_ = 0 mS/cm^2^. Inhibitory stimulations (blue triangles) are applied: (b) CTD 0 ms. No PIF; (c) CTD 2 ms. PIF; (d) CTD 5 ms. No PIF.

Similarly to Ref [2], the stimulations with random ICIs are studied, as shown in Figures 5–7. For type I and II excitabilities, the time duration of the stimulation series is 13000 ms, which contains 50 pairs of the stimulations. The ICI values follow the Poisson distribution with *λ* = 245. Due to the subthreshold oscillations for type III excitability are fast, a short time duration of 50 pairs of stimulation series 1000 ms is used, and the ICI values follow the Poisson distribution with *λ* = 19.17. The CTD is fixed in each pair of the inhibition and excitation stimulation. The stimulation intensity is set to be *G*_ex_ = 1 mS/cm^2^ for type I excitability, *G*_ex_ = 1.1 mS/cm^2^ for type II excitability, and *G*_ex_  = 2.5 mS/cm^2^ for type III excitability.

For type I excitability, the results before 6500 ms are shown in Figure 5. Seven out of 26 stimulation pulses (red triangles) induce action potentials, as shown in Figure 5(a). After the inhibitory stimulations (blue triangles) with *G*_inh_ = 0.8 mS/cm^2^ are applied, the action potentials depicted in Figure 5(a) are suppressed, as shown in Figures 5(b)–5(d). For CTD 0 ms, the 7 action potentials are suppressed to disappear, as shown in Figure 5(b), which shows that no PIF phenomenon is evoked. The result for CTD 40 ms resembles that of CDT 0 ms, as depicted in Figure 5(c). For CTD 80 ms, the inhibitory stimulations still suppress the action potentials shown in Figure 5(a), and only 4 action potentials are evoked, as depicted in Figure 5(d). The results imply that the PIF phenomenon is not evoked by the random stimulations for type I excitability.

The results (1270-4700 ms) for type II excitability are depicted in Figure 6. Six out of 14 excitatory stimulation pulses induce action potentials, as shown in Figure 6(a). After introducing inhibitory stimulations (blue triangles, *G*_inh_ = 1.4 mS/cm^2^), the results are illustrated in Figures 6(b)–6(d). For CTD 0 ms, i.e., that each inhibitory stimulation is applied at the same timing as each excitatory stimulation, the action potentials are inhibited to disappear, as shown in Figure 6(b), which shows no PIF phenomenon for CTD 0 ms. However, the inhibitory stimulations with CTD 40 ms can induce 11 spikes, as depicted in Figure 6(c), showing that the PIF phenomenon is evoked for CTD 40 ms. For CTD 80 ms, no spikes are evoked, and the PIF phenomenon disappears, as illustrated in Figure 6(d). The results imply that the PIF phenomenon can be evoked by the proper CTD values for type II excitability.

The results for type III excitability resemble those of type II excitability, as shown in Figure 7. For the excitatory stimulations induced-action potentials (Figure 7(a)), application of the inhibitory stimulations with proper strength (*G*_inh_ = 0.5 mS/cm^2^) and application timing (CTD values) can facilitate the action potentials, such as CTD = 2 ms, as illustrated in Figure 7(c). However, for other application timing (CTD values) of the inhibitory stimulations, the PIF phenomenon cannot be evoked, for example, CTD = 0 ms and 5 ms, as illustrated in Figures 7(b) and 7(d), respectively.

### 3.2. The Changes of CTD for the PIF Phenomenon

#### 3.2.1. The Changes of CTD with Respect to *G*_inh_

**Figure 8:**
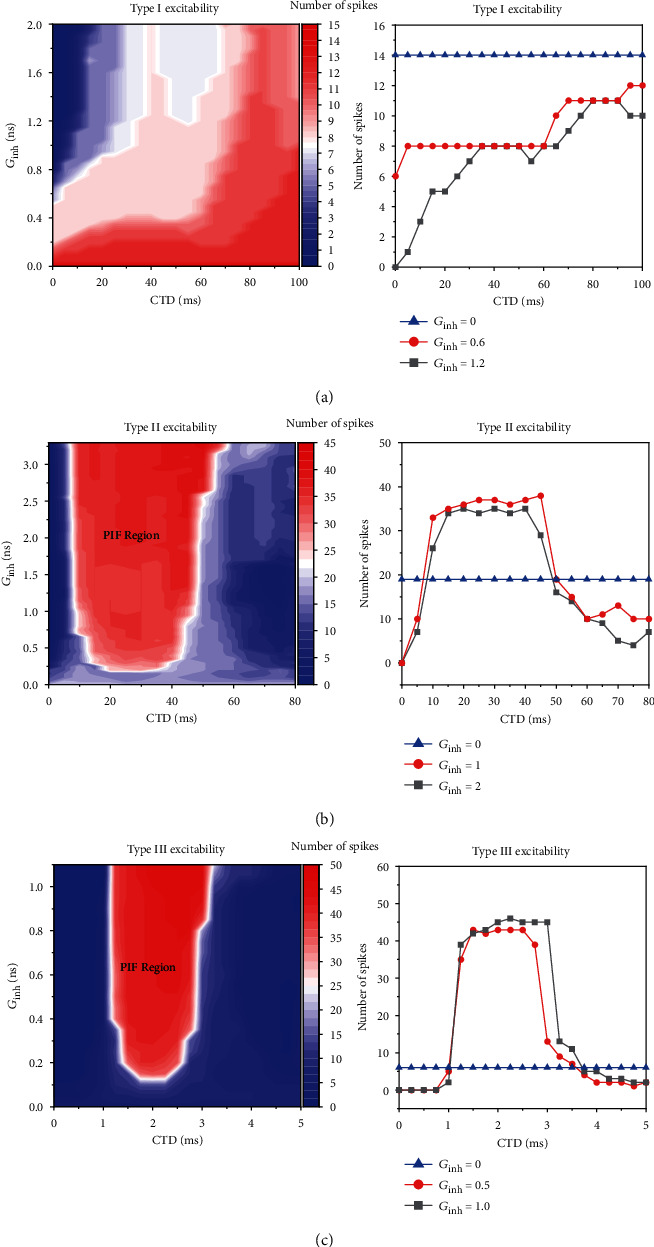
The dependence of the firing rate on both strength (*G*_inh_) and application timing (CTD) of the inhibitory stimulation for 3 types of excitability: (a) the firing rate on the plane (CTD, *G*_inh_); (b) the changes of the firing rate with increasing CTD at different *G*_inh_ values. Blue curve represents *G*_inh_ = 0 mS/cm^2^. (a1, a2) Type I excitability. *G*_inh_ = 0.6 (red) and 1.2 mS/cm^2^ (black) for *G*_ex_ = 1 mS/cm^2^ in (b). (b1, b2) Type II excitability. *G*_inh_ = 0.6 (red) and 1.2 mS/cm^2^ (black) for *G*_ex_ = 1.1 mS/cm^2^. (c1, c2) Type III excitability. *G*_inh_ = 0.5 (red) and 1.0 mS/cm^2^ (black) for *G*_ex_ = 2.5 mS/cm^2^.

The detailed dependence of the firing rate (spike number within 13000 ms) on *G*_inh_ for 3 types of excitbaility is shown in Figure 8.

For type I excitability (*G*_ex_ = 1  mS/cm^2^), with increasing the strength of inhibitory stimulation, *G*_inh_, the spike rate for all CTD values decreases (from red to blue), as shown in Figure 8(a1). The changes of the firing rate with increasing CTD values at different *G*_inh_ values are shown in Figure 8(a2). Red and black curves in Figure 8(a2) represent the spike rates for *G*_inh_ = 0.6 and 1.2 mS/cm^2^, respectively, and the blue curve represents the spike rate for *G*_inh_ = 0 mS/cm^2^. Both spike rates for *G*_inh_ = 0.6 and 1.2 mS/cm^2^ are lower than that of *G*_inh_ = 0 mS/cm^2^, and the spike rate for *G*_inh_ = 0.6 mS/cm^2^ is larger than that of *G*_inh_ = 1.2 mS/cm^2^, which shows that the stronger the inhibitory stimulations, the larger the inhibition effectiveness of the inhibitory stimulations. The largest decrease of firing rate occurs when CTD is around 0 ms. Therefore, the PIF phenomenon cannot be evoked for type I excitability.

For type II excitability (*G*_ex_ = 1.1 mS/cm^2^), the spike rate distributions are shown in Figure 8(b1). The spike rate becomes larger with increasing *G*_inh_ in the region labeled by the red, i.e., the PIF window. With increasing *G*_inh_, the left border of the PIF window becomes shorter slightly, and the right border becomes larger to a small extent. The PIF window becomes wider with increasing *G*_inh_. Within the PIF window, the spike rate increases with increasing *G*_inh_. In the blue region with small CTD or large CTD, i.e., the left and right sides to the PIF window, the spike rate becomes less with increasing *G*_inh_. Such results can be found from Figure 8(b2). The red and black curves in Figure 8(b2) represent the spike rate for *G*_inh_ = 1 and 2 mS/cm^2^, respectively, and the blue curve represents the spike rate for *G*_inh_ = 0 mS/cm^2^. For *G*_inh_ = 1 mS/cm^2^, the spike rates for CTD between 10 and 47 ms (PIF window is 47 − 10 = 37 ms) are larger than those of *G*_inh_ = 0 mS/cm^2^. For *G*_inh_ = 2 mS/cm^2^, the PIF window gets slightly wider, resulting in a range from 9 to 50 ms (50 − 9 = 41 ms). And the average number of spikes increases from around 33 for *G*_inh_ = 1 mS/cm^2^ to 36 for *G*_inh_ = 2 mS/cm^2^. Outside of the PIF window, the spike rate decreases. The results show that the width of the PIF window becomes slightly wider with increasing *G*_inh_ and still approximates half period of the subthreshold oscillations (*T*_1_/2 = 40 ms), as shown in both Figure 8(b1 and b2).

For type III excitability (*G*_ex_ = 2.5 mS/cm^2^), the PIF phenomenon shows the characteristic similar to that of type II excitability in quality, as shown in Figure 8(c1 and c2). However, the PIF phenomenon shows the characteristic different from that of type II excitability in quantity. The PIF window becomes much shorter and narrower. For example, the PIF window is CTD within 1 ms and 3.7 ms for *G*_inh_ = 1.0 mS/cm^2^ and CTD within 1 ms and 3.5 ms for *G*_inh_ = 0.5 mS/cm^2^, as shown in Figure 8(c1) and (c2).

#### 3.2.2. The Dependence of the PIF Phenomenon on *G*_inh_ at Different *G*_ex_ Values for Type III Excitability

**Figure 9:**
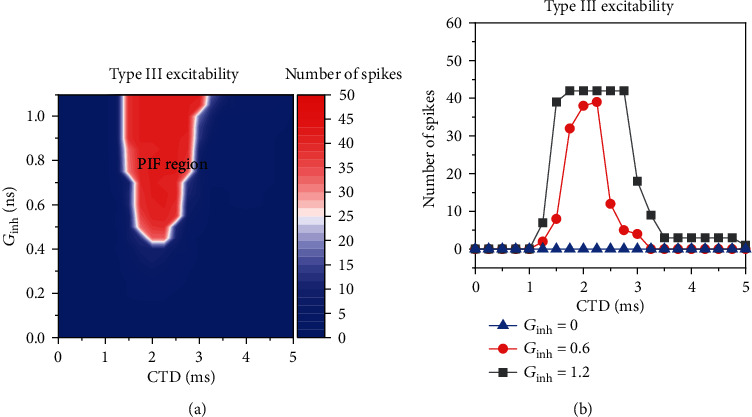
The dependence of the PIF phenomenon on *G*_inh_ and CTD for type III excitability when *G*_ex_ = 1.5 mS/cm^2^. (a) The firing rate on the parameter plane (CTD, *G*_inh_); (b) the changes of the firing rate with increasing CTD at different *G*_inh_ values. Blue, red, and black curves represent *G*_inh_ = 0, 0.6, and 1.2 mS/cm^2^, respectively.

Compared with type II excitability, the PIF window for type III excitability is shorter and narrower, which is closer to the experiment with CTD in scale of hundreds of microsecond [1]. Therefore, the PIF phenomenon for type III excitability is further studied. For *G*_ex_ = 1.5 mS/cm^2^, which is smaller than that of Figure 8(c1), the dependence of the PIF phenomenon on *G*_inh_ = 0.5 mS/cm^2^ is shown in Figures 9(a) and 9(b). Compared with Figure 8(c1), the *G*_inh_ value to evoke the PIF phenomenon becomes larger, and the PIF window becomes narrower, as shown in Figure 9(a); compared with Figure 8(c2), the spike rate for the PIF phenomenon becomes slightly smaller, as shown in Figure 9(b). Especially, the PIF window for *G*_ex_ = 1.5 mS/cm^2^ and *G*_inh_ = 0.5 mS/cm^2^ is narrow, which shows that the PIF window is dependent on both *G*_ex_ and *G*_inh_ to a certain extent. The *G*_ex_ is smaller, or the  *G*_inh_ is smaller; the PIF window becomes narrower. Therefore, the width of the PIF window can cover half the period of the subthreshold oscillations when *G*_ex_ and  *G*_inh_ are relatively large and becomes narrower as *G*_ex_ or  *G*_inh_ becomes relatively weaker.

### 3.3. Threshold Mechanism for the PIR Phenomenon

#### 3.3.1. Threshold Curve for Action Potential Evoked from the Steady State

**Figure 10:**
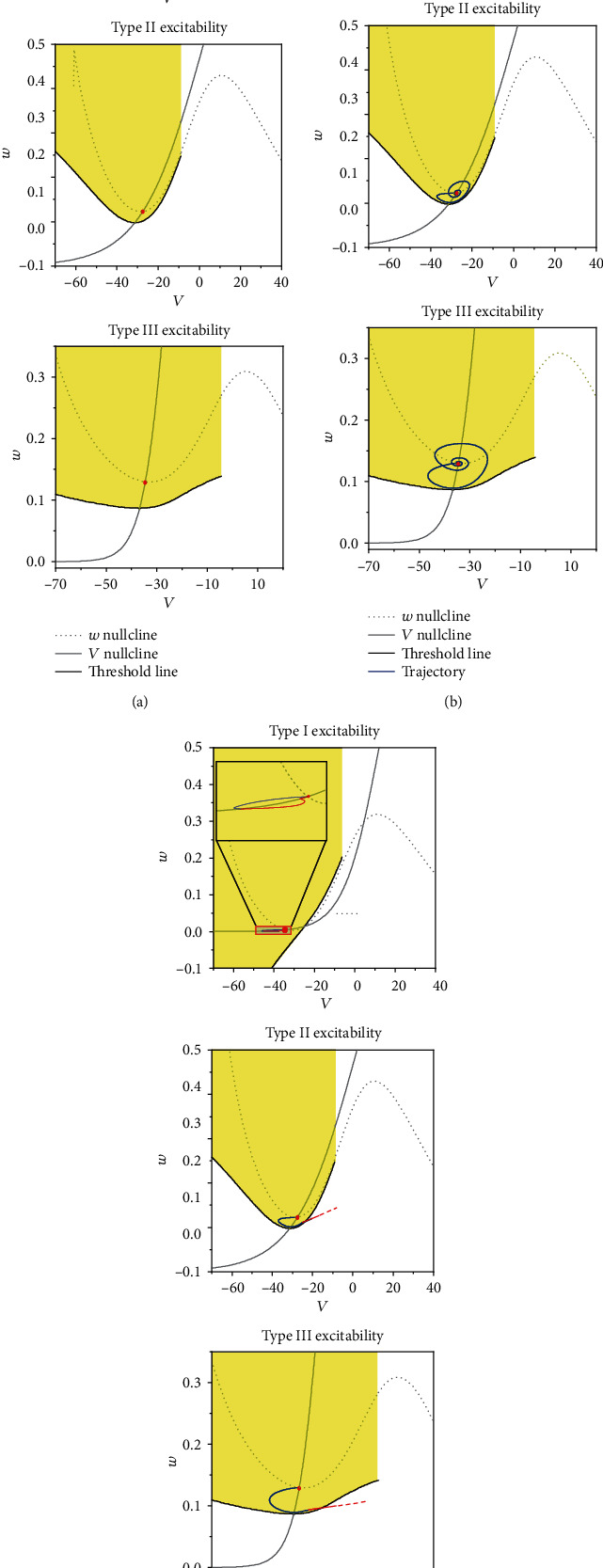
The dynamics for 3 types of excitability: (a) the threshold curve (black solid curve), the *w* nullcline (gray solid curve), the *V* nullcline (grey dotted curve), and the stable equilibrium point (red circle); (b) phase trajectory (red) of the subthreshold oscillations induced by the inhibitory stimulation plotted with the (a); (c) an excitatory stimulation is applied at a phase point in the ascending part of the subthreshold oscillations (the initiation point of the red curve). (a1, a2, a3) Type I excitability for *I*_app_ = 38 *μ*A/cm^2^; *G*_inh_ = 1 mS/cm^2^; *G*_ex_ = 1.1 mS/cm^2^; insert in (a3) represents the enlargement of the phase trajectory. (b1, b2, b3) Type II excitability for *I*_app_ = 87.3 *μ*A/cm^2^; *G*_inh_ = 0.5 mS/cm^2^; *G*_ex_ = 1 mS/cm^2^. (c1, c2, c3) Type III excitability for *I*_app_ = 200 *μ*A/cm^2^; *G*_inh_ = 0.6 mS/cm^2^; *G*_ex_ = 2.5 mS/cm^2^.

Except for the different dynamics in bifurcations for 3 types of excitability [10, 12, 16, 17], the thresholds to evoke an action potential from steady state for 3 types of excitability are different, as shown in Figure 10. The threshold curve in the phase plane (*V*, *w*) is acquired as follows: Each phase point (*V*, *w*) (the interval between *V* is 0.1 mV, and the interval between *w* is 0.001) is assigned to the initial values of the ML model without stimulation (Eqs. (1) and (2)). If the initial value corresponding to a phase point can induce an action potential, the location of the phase point in the phase plane is labelled by the yellow; if the initial value cannot evoke an action potential, the location of the phase point corresponding to the initial value is labelled by the white. The border between the yellow and white area forms the threshold curve in the phase plane (*V*, *w*), as shown in Figure 10. Therefore, if the phase point (*V*, *w*) in the white area is assigned to be the initial values of the ML model, an action potential appears. However, the phase point (*V*, *w*) in the yellow area chosen as the initial values cannot induce an action potential. The nullclines d*V*/*dt*  = 0 and d*w*/*dt* = 0 are represented by the grey solid and dashed curves. The red bold circle represents the steady state, and the blue curve represents the trajectory after an inhibitory stimulation. It should be noticed that the white and yellow areas around the steady state are acquired in the present paper and far from the steady state (the up-right corner) are not calculated (no relevance to the results of the present paper).

For type I excitability with *I*_app_ = 38 *μ*A/cm^2^, the behavior of the ML model is the steady state corresponding to a stable node (red dot), which is the left intersection point between the two nullclines, as shown in Figure 10(a1 and a2). The threshold curve exhibits a positive slope and locates right to the stable node. The blue trajectory induced by the inhibitory stimulation locates left to the stable node. Compared with the stable node, the distance of the blue trajectory to the threshold curve becomes larger, which shows that the inhibitory stimulation enhances the stimulation strength to evoke an action potential from the behavior after the inhibitory stimulation. Therefore, an excitatory stimulation, which cannot induce an action potential from the steady state, still cannot induce the trajectory runs across the threshold curve, as shown by the red curve in Figure 10(a3) and the insert figure of Figure 10, which is the enlargement of the trajectory. All trajectories locate within the yellow area; therefore, the PIF phenomenon cannot be evoked.

When *I*_app_ = 87.3 *μ*A/cm^2^, the behavior of the ML model for type II excitability is the steady state (a stable focus, red dot), which is the unique intersection point between the two nullclines, as shown in Figure 10(b1 and b2). The threshold curve exhibits a shape very different from that of type I excitability, which exhibits an “U”-like shape with a negative slope for the part left to the stable focus and a positive slope for the part right to the stable focus, similar to the threshold sets in Ref [12]. The stable focus is slightly upper to the bottom part of the threshold curve. The behavior evoked by an inhibitory stimulation exhibits subthreshold oscillations around the stable focus, as shown by the blue curve in Figure 10(b2). The blue trajectory starts from the steady state (red point), rotates in anticlockwise, and at last recovers to the steady state. The strength of an excitatory stimulation to evoke an action potential from the suitable phase of the subthreshold oscillations (blue curves) becomes less than the one from the stable focus. Therefore, an excitatory stimulation, which cannot induce an action potential from the steady state, can induce the trajectory run across the threshold curve (red curve) to form an action potential (not shown here because too large amplitude), as shown in Figure 10(b3). The excitatory stimulation is applied at the ascending process of the subthreshold oscillations; the membrane potential can run across the threshold curve at a phase down-left to the steady state, as depicted by the red curve in Figure 10(b3), which is the cause for the PIF phenomenon.

For type III excitability, when *I*_app_ = 200 *μ*A/cm^2^, the behavior of the ML model is the steady state corresponding to the stable focus (red dot), which is the unique intersection point between the two nullclines, as shown in Figure 10(c1 and c2). The threshold curve and the subthreshold oscillations evoked by the inhibitory stimulation resemble those of type II excitability, as illustrated in Figure 10(c2). Therefore, at a suitable phase of the oscillations, an excitatory stimulation can induce the PIF phenomenon, as shown by the red curve in Figure 10(c3).

#### 3.3.2. Inhibitory Stimulation Induces Subthreshold Oscillations and the Spontaneous Threshold

**Figure 11:**
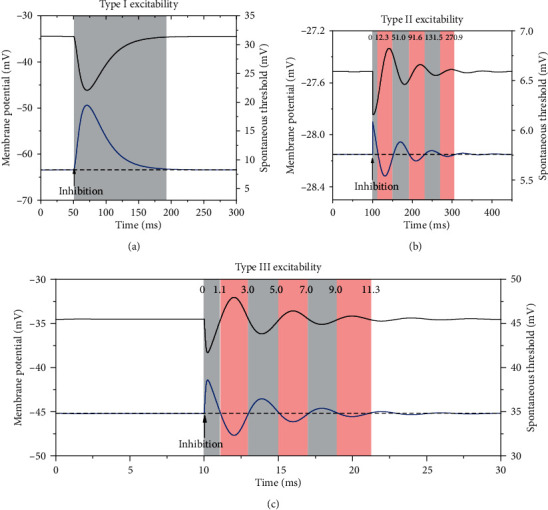
The subthreshold oscillations (black) induced by an inhibitory stimulation and the corresponding “spontaneous threshold” (blue). (a) Type I excitability. Inhibition stimulation starts from *t* = 50 ms. *G*_inh_ = 1 mS/cm^2^. (b) Type II excitability. The inhibition stimulation starts at *t* = 100 ms. *G*_inh_ = 3.1 S/cm^2^ and *τ*_inh_ = 0.05 ms. (c) Type III excitability. The inhibition stimulation starts at *t* = 10 ms. *G*_inh_ = 1.1 mS/cm^2^ and *τ*_inh_ = 0.05 ms. The pink windows correspond to the lower spontaneous threshold and represent the PIF window.

In the present subsection, the ability for the subthreshold oscillations from which an action potential can be evoked is investigated. As can be found from the middle panels of Figure 10, the distances between each phase point in the phase plane to the threshold curve right to the phase point can be used to measure the ability to evoke an action potential from the phase point by the excitatory stimulation. In general, if the distance is small, it is easy that an action potential can be evoked by an excitatory stimulation, i.e., that the PIF phenomenon is easy to be evoked. Therefore, the distance from a phase point (*V*(*t*), *w*(*t*)) to the threshold curve is defined as a “Spontaneous Threshold” *V*_*T*_(*t*), which is described as follows:

(4) $$\begin{array}{c} \begin{array}{c} V_{T}\left(t\right)=\left|V_{\text{threshold}}-V\left(t\right)\right|, \end{array} \end{array}$$

where *V*(*t*) is the membrane potential of the subthreshold oscillations at timing *t* and *V*_threshold_ is the *V* value of the point in the threshold curve with the same value *w*(*t*) as the phase point. The spontaneous threshold *V*_*T*_(*t*) describes the horizontal Euclidean distance between a phase point on the phase trajectory and the threshold curve. The detailed changes of *V*(*t*) and *V*_*T*_(*t*) are depicted by the black and blue curves in Figure 11.

For type I excitability, the spontaneous threshold *V*_*T*_(*t*) after the inhibition stimulation (*t* = 50 ms) is not smaller than that of the steady state, as shown by blue curve in Figure 11(a), which implies that the PIF phenomenon cannot be evoked. In fact, *V*_*T*_(*t*) is larger than that of the steady state within a relatively long duration after the inhibitory stimulation, which shows that the firing activity should be suppressed. After the application of the inhibition stimulation, to evoke an action potential becomes increasingly difficult.

For type II excitability, the spontaneous threshold *V*_*T*_(*t*) (blue curve) after the inhibition stimulation (*t* = 100 ms for the lower horizontal ordinate) manifests the damping oscillations with the same intrinsic period as that of the subthreshold oscillations *V*(*t*) (the black curve), as shown in Figure 11(b). During the middle and ending phase of the ascending part and the beginning phase of the descending part within a period of the subthreshold oscillations of *V*(*t*), i.e., during the pink windows, *V*_*T*_(*t*) is lower than that of the steady state (the horizontal dashed line). Therefore, it is easy to evoke the PIF phenomenon during these windows, which correspond to the PIF windows. Each PIF window approximates half of the intrinsic period of the subthreshold oscillations. The first PIF window for type II excitability is from 12.3 to 51 ms (the upper horizontal ordinate), which is in accordance with the PIF window (8.5 ms, 51.5 ms) in Figure 2 to a large extent. The slight difference of the PIF window between Figures 3(b) and 11(b) is due to the distinct parameters of the synapses. For example, *τ*_inh_ = 1 ms in Figure 3(b) and *τ*_inh_ = 0.05 ms for Figure 11(c).

For type III excitability, the spontaneous threshold *V*_*T*_(*t*) (blue curve) after the inhibition stimulation (*t* = 10 ms, the lower horizontal ordinate) manifests dynamics similar to that of type II excitability, as shown in Figure 11(c). The first PIF window is from 1.1 to 3.0 ms, which is in consistent with the PIF window in Figure 3(c) (1.1 ms, 3.1 ms) to a large extent.

#### 3.3.3. Different Bifurcations for Type I, II, and III Excitabilities

**Figure 12:**
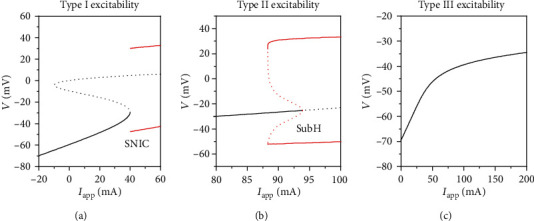
Bifurcations for three types of excitability. Black solid curve represents the stable steady state, black dotted curve represents the unstable steady state, red solid curves represent stable limit cycle, and red dotted curves represent the unstable limit cycle. (a) Type I excitability. The SNIC represents the saddle-node bifurcation on an invariant cycle. (b) Type II excitability. The SubH represents the subcritical Hopf bifurcation. (c) Type III excitability. No bifurcations or stable firing appears.

In fact, the different dynamics for the threshold curve is determined by the types and bifurcations of the equilibrium point. With changing parameter such as *I*_app_, the steady state or equilibrium point can change to firing via three types of excitability, type I, type II, and type III, as shown in Figure 12. In nonlinear dynamics, type I excitability corresponds to saddle-node bifurcation on an invariant cycle (SNIC), type II to a subcritical Hopf bifurcation (SubH), and type III to phasic firing without bifurcation, as shown in Figures 12(a)–12(c), respectively.

For type I excitability illustrated in Figure 12(a), the left black solid line represents the steady state corresponding to the stable node, the middle and upper dashed curves correspond to saddle and unstable equilibrium point, respectively, and the upper and lower solid red lines represent the maximal and minimal values of the membrane potentials, respectively. The SNIC represents the saddle-node bifurcation on an invariant cycle, which appears at *I*_app_ ≈ 39.96 *μ*A/cm^2^.

For type II excitability depicted in Figure 12(b), the left black solid line represents the steady state corresponding to the stable focus, and the upper and lower solid red curves represent the maximal and minimal values of the membrane potentials, respectively. The SubH represents a subcritical Hopf bifurcation point at *I*_app_ ≈ 93.8 *μ*A/cm^2^. The intersection point between the dashed (unstable limit cycle) and bold red curves represents a fold bifurcation of the limit cycles at *I*_app_ ≈ 88.3 *μ*A/cm^2^.

No bifurcations or stable firing behaviors appear for type III excitability, as depicted in Figure 12(c). In the present paper, more detailed descriptions of the bifurcations for 3 types of excitability are not described, which can be found in the previous investigations [15, 16].

## 4. Conclusions

The postinhibitory facilitation (PIF) of firing in the auditory system observed in the biological experiments is very important for both fundamental conception and biological significance of neuroscience [1–3]. On the one hand, it has been related to the sound location or coincidence detection. On the other hand, a counterintuitive or paradoxical function of the inhibitory modulation is present. In general, the inhibitory modulation always plays a role to inhibit the firing activity [6–8]. However, the PIF phenomenon presents that the inhibitory modulations can facilitate the firing activity, which extends the functions of the inhibitory modulations. In the present paper, the excitability and threshold mechanisms for the PIF phenomenon are acquired in a theoretical model, which present comprehensive and deep explanations to the PIF phenomenon. The progress or novelty exhibits in the following three aspects.

Firstly, in the present paper, the PIF phenomenon is built a relationship to type II and III excitabilities instead of type I excitability. The PIF phenomenon for type III excitability exhibits a CTD range shorter than that of type II excitability, which is closer to the experimental observations to a large extent [1]. Therefore, the PIF phenomenon in the auditory system may correspond to type III excitability, which is consistent with other Refs [27–32].

Secondly, the threshold mechanisms of the PIF phenomenon for type II and III excitabilities are acquired in the present paper. The threshold curve for type I excitability is different from those of type II and III excitabilities. For type II and III excitabilities, there is a part of the threshold curve locating left to the steady state and a part lower to the steady state, which is similar to the threshold sets for type II excitability in Ref [12] and the threshold curve in Ref [2]. Such left and lower parts of the threshold curve are the intrinsic cause for the PIF phenomenon.

Last, a theoretical estimation to the range of CTD (interval between inhibitory and excitatory stimulations) for the PIF phenomenon, i.e., the PIF window, is acquired to be related to the intrinsic period of the subthreshold oscillations. If the stimulations are relatively strong, the PIF window approximates half period of the subthreshold oscillations. If the stimulations are relatively small, the PIF window becomes narrower. Such an estimation for the PIF window may be helpful for the choice of CTD in the experiment.

In the present paper, the dynamical mechanism such as excitability and threshold mechanism for the PIF phenomenon in a single neuron is investigated. Based on the theoretical viewpoint of the present paper, in the future, the PIF phenomenon should be studied in the following aspects. First, considering the importance of synapse [47], the dependence of the PIF phenomenon on the synaptic parameters should be studied, which has been put less attention than the PIF window and the synaptic conductance in both the previous studies [2, 3] and the present paper. The parameter values of the synaptic parameters are chosen according to Ref [2] and different dynamics of 3 types of excitability. The values of *E*_ex_ = −10 mV and *E*_inh_ = −66.5 mV for type I and type II excitabilities are the same as those in Ref [2]. For type II excitability,  *τ*_ex_ = 3 ms and *τ*_inh_ = 1 ms are chosen as values approximating one-tenth of the intrinsic period of the firing or subthreshold oscillations (about tens of milliseconds). For type I excitability, the period of the firing near the bifurcation point becomes very long; therefore, one parameter is chosen as a value larger (*τ*_inh_ = 10 ms) than that of type II excitability (*τ*_inh_ = 1 ms), and the other is assigned to be the same value as that of type II excitability (*τ*_ex_ = 3 ms). For type III excitability, *E*_ex_ = −10 mV, which is the same as those of Ref [2] and of type I and type II excitabilities. The type III exhibits a short period of the subthreshold oscillations; therefore, *τ*_ex_ = 0.25 ms, which is shorter than that of type II excitability, and *τ*_inh_ = 1 ms, which is the same as that of type II excitability, are chosen. Due to the changes of the value of the time constant, *τ*_inh_, the *E*_inh_ is changed to -96.5 mV for type III excitability. In the future, the changes of the PIF phenomenon with respect to the changes of the synaptic parameters should be investigated in detail. Secondly, the dependence of the PIF phenomenon on the ionic current (such as the hyperpolarization active caution current and a low-threshold potassium current) should be studied to build a close relationship to the experimental observations in the auditory system [1, 3]. Last, except for the dynamical mechanism for the PIF phenomenon in the single neurons, the physiological roles for the PIF phenomenon in the auditory system should be studied.

## Figures and Tables

**Table 1:** Parameter values for 3 types of excitability Morris-Lecar model.

	Type I	Type II	Type III
*g*_Ca_ (mS/cm^2^)	4.4	4	20
*g*_*K*_ (mS/cm^2^)	8	8	20
*g*_*L*_ (mS/cm^2^)	2	2	2
*E*_Ca_ (mV)	120	120	50
*E*_*K*_ (mV)	-84	-84	-100
*E*_*L*_ (mV)	-60	-60	-70
*V*_1_ (mV)	-1.2	-1.2	-1.2
*V*_2_ (mV)	18	18	18
*V*_3_ (mV)	2	12	-25
*V*_4_ (mV)	30	17.4	10
*C* (*μ*F/cm^2^)	20	20	2
*ϕ*	0.04	0.067	0.15
*I*_app_ (*μ*A/cm^2^)	38	87.3	200

## Data Availability

The simulation and analysis data used to support the findings of this study are available from the corresponding author upon request.
